# Editorial: Inflammatory responses on the road from NASH to HCC: pathogenic mechanisms and possible therapeutic strategies

**DOI:** 10.3389/fimmu.2024.1512363

**Published:** 2024-11-01

**Authors:** Veronika Lukacs-Kornek, Tim Hendrikx, Salvatore Sutti

**Affiliations:** ^1^ Institute of Molecular Medicine and Experimental Immunology, University Hospital Bonn of the Rheinische Friedrich-Wilhelms-University, Bonn, Germany; ^2^ Department of Laboratory Medicine, Medical University Vienna, Vienna, Austria; ^3^ Department of Health Sciences, University of Eastern Piedmont, Novara, Italy

**Keywords:** MASLD, MASH, NASH, inflammation, immunity, liver

Non-alcoholic fatty liver disease (NAFLD) was recently renamed to metabolic dysfunction-associated liver disease (MASLD) to emphasize the metabolic dysfunctions that most often accompany its manifestation (1). MASLD covers a spectrum of liver diseases, ranging from simple steatosis to metabolic dysfunction-associated steatohepatitis (MASH), a clinical condition characterized by steatosis, hepatocellular injury, and inflammation with or without fibrosis (1). MASH can further progress to cirrhosis and, in a certain fraction of patients, to hepatocellular carcinoma (HCC) (2). Multiple factors sustain the transition from MASLD to MASH and its advanced stages, but emerging evidence indicates chronic inflammation as the driving force underlying the process (2). Therefore, understanding the cellular and molecular mechanisms responsible for inflammatory responses may lay the foundations for treatments to counteract MASH and its complications.

In this article Research Topic, the authors summarized the recent advances concerning the modulation of inflammatory responses associated with MASH.

Growing evidence indicates that hepatokines mediate key steps of MASLD/MASH (3, 4). Foglia et al. addressed this issue by exploring the pathological implications of the histidine-rich glycoprotein (HRG) in MASH-related carcinogenesis. HGR is a glycoprotein which interacts with several molecules and modulates essential biological processes (5). The authors found that HRG levels increase in parallel with the disease severity, and the HRG genetic deletion protects mice from MASH-related HCC by affecting multiple pathways.

Hepatokines, like other molecules, mediate biological effects by triggering specific receptors, which transduce complex signals underlying MASH (6). Apostolo et al. reviewed the implications of TAM receptors (Tyro 3, Axl and MerTK) in the transition from MASLD/MASH to HCC. The authors described that TAM receptors, along with the common ligand Growth Arrest Specific (GAS) 6, support the development of MASH. Indeed, the Gas6/TAM axis modulates inflammatory responses and stimulates the activation, survival and proliferation of hepatic stellate cells (HSCs). The TAM/Gas6 system also promotes the induction of signalling pathways involved in tumour growth and dissemination. Furthermore, it may lead to resistance to sorafenib in HCC cells, so much so that pharmacological targeting of Axl restores the cell sensitivity to sorafenib. Grøndal et al. further investigate TAM receptors in MASH, observing that the levels of the soluble form of Axl (sAxl) increase in preclinical models of MASLD/MASH, in parallel with the induction of inflammatory responses, suggesting that sAxl may represent an early marker for MASLD/MASH. The authors also described the mechanistic involvement of Axl in MASLD/MASH, reporting that its pharmacological inhibition reduces steatosis, inflammation and fibrosis. The authors also investigated the mechanisms by which Axl inhibition ameliorates MASLD/MASH, describing that Axl inhibition has an immunomodulatory action by lowering the hepatic expression of pro-inflammatory mediators along with the abundance of NK cells and various dendritic cell (DC) subsets.

Besides TAM, additional families of receptors contribute to inflammatory responses like protease-activated receptors (PARs). PARs are the substrate of multiple proteases capable of cutting the extracellular N-terminus sequence, which becomes the agonist of PARs themselves (7). Villano and Pontisso reviewed the current available data on PAR2 and its implications in MASLD/MASH. PAR2 activation drives MASLD development by impairing the insulin pathway and causing insulin resistance (IR) and liver steatosis. PAR2 also promotes the transition from MASLD to MASH by activating inflammatory pathways and HSCs. PAR2 activation also supports cell transformation by inhibiting the AMPK-mediated autophagy and promotes tumour growth and metastatization *via* the PI3K/AKT signaling pathway. Consistently, PAR2 antagonists inhibit liver injury, inflammation and fibrosis in the experimental MASH.

MASH-associated inflammatory responses involve multiple immune cell subsets belonging to the innate and adaptive immunity branches, such as macrophages (MFs), dendritic cells (DCs), NK/NKT cells and T/B-lymphocytes (8–10). These cells influence the hepatic microenvironment by secreting multiple cyto/chemokines and mediating the disease progression (11). Saueret al. analyzed this issue by investigating the effects of the secretome derived from differentially polarized human primary monocytes/macrophages and NK cells in activating HSCs. The authors found that “classical” stimuli for MFs (TNF-α, IL-4 and IL13) and NK cells (IL-15) fail in activating HSCs *in vitro*. Conversely, the hyperactivation of MFs and NK cells with the phorbol 12-myristate 13-acetate (PMA), a non-specific pro-inflammatory agent, promotes a strong activation of HSCs. Overall, these results reflect the complex nature of the inflammatory and fibrogenic responses and suggest that other factors may act *in vivo* in activating HSCs.

Pinto and Lukacs-Kornek analyzed the complexity of inflammatory responses in MASLD/MASH by reviewing recent advances in the contribution of DCs to the disease. The authors summarized the multiple mechanisms by which DCs may support hepatic inflammation in MASH. They emphasized the capacity of DCs to interact with further immune cells and initiate adaptive immune responses by acting as antigen-presenting cells for both T and B-lymphocytes. However, they also have brought to light the controversial nature of currently existing data related to the involvement of DCs in MASLD/MASH, so much so that the precise contribution of DCs to MASLD/MASH remains poorly defined, and further investigations are needed.

The induction of innate and adaptive immune responses represents a crucial pathological mechanism in MASLD/MASH (12). However, the mechanisms governing the reciprocal interactions between innate and adaptive immunity are still poorly characterized. Provera et al. investigated the role of the costimulatory molecules ICOS and its ligand (ICOS-L), finding a possible role for such molecules in the crosstalk between CD8^+^T-lymphocytes and TREM2^+^ monocyte-derived macrophages (MoMFs) and then in MASH. They observed that the disruption of the ICOS/ICOS-L system improves histological features of MASH by lowering the faction of TREM2^+^ MoMFs. Overall, these results suggest that the crosstalk between CD8^+^T-lymphocytes and TREM2^+^ MoMFs mediated by the ICOS/ICOS-L dyad supports a pool of TREM2^+^ MoMFs, contributing to MASH.

Altogether, the data discussed in the present Research Topic summarize and elaborate on mechanisms responsible for the induction and progression of inflammatory responses in MASH. They also corroborated the view that chronic inflammation is a critical player in MASLD/MASH progression. Finally, they highlighted potential biomarkers and pharmacological targets exploitable for fighting MASH.

## References

[1] Rinella ME Lazarus JV Ratziu V Francque SM Sanyal AJ Kanwal F. A multisociety Delphi consensus statement on new fatty liver disease nomenclature. J Hepatol. (2023) 79:1542–56. doi: 10.1016/j.jhep.2023.06.003 37364790

[2] Provera A Vecchio C Sheferaw AN Stoppa I Pantham D Dianzani U. From MASLD to HCC: what’s in the middle? Heliyon. (2024) 10:e35338. doi: 10.1016/j.heliyon.2024.e35338 39170248 PMC11336632

[3] Stefan N Schick F Birkenfeld AL Häring HU White MF. The role of hepatokines in NAFLD. Cell Metab. (2023) 35:236–52. doi: 10.1016/j.cmet.2023.01.006 PMC10157895 36754018

[4] Cannito S Foglia B Villano G Turato C Delgado TC Morello E. SerpinB3 differently up-regulates hypoxia inducible factors -1α and -2α in hepatocellular carcinoma: mechanisms revealing novel potential therapeutic targets. Cancers (Basel). (2019) 11:1933. doi: 10.3390/cancers11121933 31817100 PMC6966556

[5] Gao S Nishibori M. Multiple roles of histidine-rich glycoprotein in vascular homeostasis and angiogenesis. Acta Med Okayama. (2021) 75:671–5. doi: 10.18926/AMO/62805 34955533

[6] Sinha RA. Targeting nuclear receptors for NASH/MASH: From bench to bedside. Liver Res. (2024) 8:34–45. doi: 10.1016/j.livres.2024.03.002 38544909 PMC7615772

[7] Heuberger DM Schuepbach RA. Protease-activated receptors (PARs): mechanisms of action and potential therapeutic modulators in PAR-driven inflammatory diseases. Thromb J. (2019) 17:4. doi: 10.1186/s12959-019-0194-8 30976204 PMC6440139

[8] Cannito S Dianzani U Parola M Albano E Sutti S. Inflammatory processes involved in NASH-related hepatocellular carcinoma. Biosci Rep. (2023) 43:BSR20221271. doi: 10.1042/BSR20221271 36691794 PMC9874450

[9] Hoebinger C Rajcic D Hendrikx T. Oxidized lipids: common immunogenic drivers of non-alcoholic fatty liver disease and atherosclerosis. Front Cardiovasc Med. (2022) 8:824481. doi: 10.3389/fcvm.2021.824481 35083304 PMC8784685

[10] Lukacs-Kornek V Schuppan D. Dendritic cells in liver injury and fibrosis: shortcomings and promises. J Hepatol. (2013) 59:1124–6. doi: 10.1016/j.jhep.2013.05.033 23727306

[11] Braunersreuther V Viviani GL Mach F Montecucco F. Role of cytokines and chemokines in non-alcoholic fatty liver disease. World J Gastroenterol. (2012) 18:727–35. doi: 10.3748/wjg.v18.i8.727 PMC3286135 22371632

[12] Sutti S Albano E. Adaptive immunity: an emerging player in the progression of NAFLD. Nat Rev Gastroenterol Hepatol. (2020) 17:81–92. doi: 10.1038/s41575-019-0210-2 31605031 PMC7222953

